# Improving the efficiency of using multivalued logic tools

**DOI:** 10.1038/s41598-023-28272-1

**Published:** 2023-01-20

**Authors:** Ibragim E. Suleimenov, Yelizaveta S. Vitulyova, Sherniyaz B. Kabdushev, Akhat S. Bakirov

**Affiliations:** 1 National Engineering Academy of the Republic of Kazakhstan, Bogenbai Batyr Str. 80, 050010 Almaty, Republic of Kazakhstan; 2 Gumarbek Daukeyev Almaty University of Power Engineering and Telecommunications, Baytursynov Str. 126/1, 050013 Almaty, Republic of Kazakhstan; 3 International Information Technology University, Manas Str, 34/1, 050013 Almaty, Republic of Kazakhstan

**Keywords:** Electrical and electronic engineering, Applied mathematics

## Abstract

Multivalued logics are becoming one of the most important tools of information technology. They are in great demand for creation of artificial intelligence systems that are close to human intelligence, since the functioning of the latter cannot be reduced to the operations of binary logic. At the same time, the problem of improving the efficiency of using the results of research in multivalued logics, as well as the problem of interpreting variables of multivalued logic, is acute. These problems create certain interdisciplinary barriers and make it difficult to implement the results of research in the field of multivalued logics in other fields of knowledge. It is shown that the problem of interpreting multivalued logic variables can be removed by establishing correspondence with fuzzy logic variables. Improving the efficiency of using of operations of multivalued logics and their variables can be provided by using their close connection to Galois fields. This connection, among other things, makes it possible to reduce any operations of multivalued logics, the number of variables in which is equal to a prime number, to algebraic functions whose arguments take values in Galois fields. This allows, among other things, to eliminate the very cumbersome constructions used in works on multivalued logic and make its apparatus convenient for use in related scientific disciplines in information technology. Direct verification of the adequacy of algorithms based on the use of Galois fields can be carried out by means of radio-electronic circuits, examples of which are presented in the present paper.

## Introduction

The emergence of non-Aristotelian logics (in particular, Lukasevich's logic^1^ N. Vasiliev's "imaginary logic"^2^) at the beginning of the twentieth century was obviously connected with the transformation of the general situation in the philosophy of mathematics and the discussions concerning the problems of justification of mathematics and logic as such^3^. As noted^2^, N. Vasiliev proposed a project of non-Aristotelian logic built without using the law of contradiction, proceeding from the analogy with the non-Euclidean geometry of N. Lobachevsky, which excludes the use of the fifth postulate of Euclid, who also initially called his geometry "imaginary". The construction of logics that partially or completely refuse to use the law of the excluded third ("every statement is either true or false", to use the simplest version of the interpretation) has subsequently led to a great variety of multivalued logics^4,5^, including paraconsistent logics^6^, paracomplete logics^7^, etc.

De facto, there are currently a huge number of varieties of multivalued logics, but the question of how exactly they are applicable to the description of the laws of thought remains open^8^.

In this respect, it is worth pointing out that, in accordance with the tradition going back to Aristotle, logic was viewed as a science of how to correctly reason, as a science of the laws of thinking. This is the way J.W. Bull interpreted it. In a famous monograph on the history of mathematics^3^ the following passage from one of J. Boole's main works, "Investigation of the laws of thought", is given to illustrate exactly this approach, which prevailed then in the field of logic creation:

"In the treatise before us we intend to investigate the fundamental laws of those operations of the mind by which thinking is effected, in order to express them in the symbolic language of calculus, and on this basis to construct the science of logic and its method."

Obviously, most modern works on multivalued logics have departed far enough from this tradition, otherwise the problem of interpretation of multivalued logics and their classification would not be so acute.

The problem of applicability of multivalued logics to the reflection of laws of thought is most closely related to the problem of interpreting the variables of multivalued logic, which remains relevant at present^9,10^. Whereas within binary logic, its variables can be uniquely associated with the notion of truth, such uniqueness is lost for multivalued logics, which determines the relevance of research in the philosophy of multivalued logics, which is currently being actively pursued^11,12^.

However, we must admit that a full-fledged return to the tradition that considers logic as a reflection of the laws of thinking, obviously, cannot be realized otherwise than on an interdisciplinary basis. This, in turn, requires overcoming pronounced interdisciplinary barriers. The language in which the works on multivalued logics are written remains difficult to comprehend for a large part of specialists in other fields of knowledge, in particular in information technologies.

A definite step towards overcoming the interdisciplinary barriers is knowingly solving the problem of visibility of variables of multivalued logics touched upon in^13,14^.

To solve this problem, it is reasonable to use the correspondence between multivalued logics and algebraic structures, such as Galois fields, which are widely used in modern information technologies, especially in cryptography^15–17^. This correspondence can be most easily established when the number of variables of a particular multivalued logic is equal to the degree of the prime number p. In this case, a Galois field element $GF({p}^{n})$ can be assigned to each value of a variable of multivalued logic in a one-to-one correspondence.

For Galois fields, in turn, the following illustrative interpretation can be proposed. As emphasized^18,19^, the standard model of a signal is a function taking values on a set of real numbers. However, in the case when the signal is reduced to a certain set of discrete levels that fit into a finite range of amplitude measurements, this approach is not mandatory. A function taking values in any finite algebraic structure, such as Galois fields, can also be used as a signal model. The simplest kind of Galois fields $GF(p)$ is formed through a homomorphism of a ring of integers to a ring of classes of deductions modulo $p$, where $p$ is a prime number.

In this paper, we show that the problem of interpreting the variables of multivalued logic can be solved, for example, by establishing a correspondence between the variables of multivalued logic and the variables of fuzzy logic. Variables of multivalued logic can also be assigned to the levels of the digitized signal in the case when the signal model is a function that takes values in Galois fields. More broadly, the variables of multivalued logic can be interpreted through the establishment of links between concepts (e.g., philosophical categories). In all these cases, it is important to have a tool that allows you to bring logical relationships to an algebraic form. For the case when the set of variables of multivalued logic can be assigned to the field $GF({p}^{n})$, this problem is solved through an analogue of the algebraic normal form presented in this paper.

Section 1 shows that the use of multivalued logic variables can be made explicit, including by mapping to multivalued logic variables.

Section "Visualization of the variables of multivalued logic" shows that for the case when the number of variables is equal to a prime number, instead of the truth tables traditionally used in works on multivalued logic, it is also possible to use an analog of the algebraic normal form (the Zhegalkin polynomial).

Section "Reduction of multivalued logic operations to algebraic ones" provides a specific example showing that multivalued logic operations can be performed using electronic devices built on typical binary logic components.

## Visualization of the variables of multivalued logic

Clear illustrations for the practical use of variables of multivalued logics are easiest to offer, focusing on the approaches used in fuzzy logic. As is known, fuzzy logic establishes a certain correspondence between ranges of continuously varying parameters and linguistic variables marking them^20^. Simplifying, the apparatus of linguistic variables allows to "transform into words" the values of parameters, which, under certain conditions, can be quantitatively measured with high accuracy.

**Figure 1:**
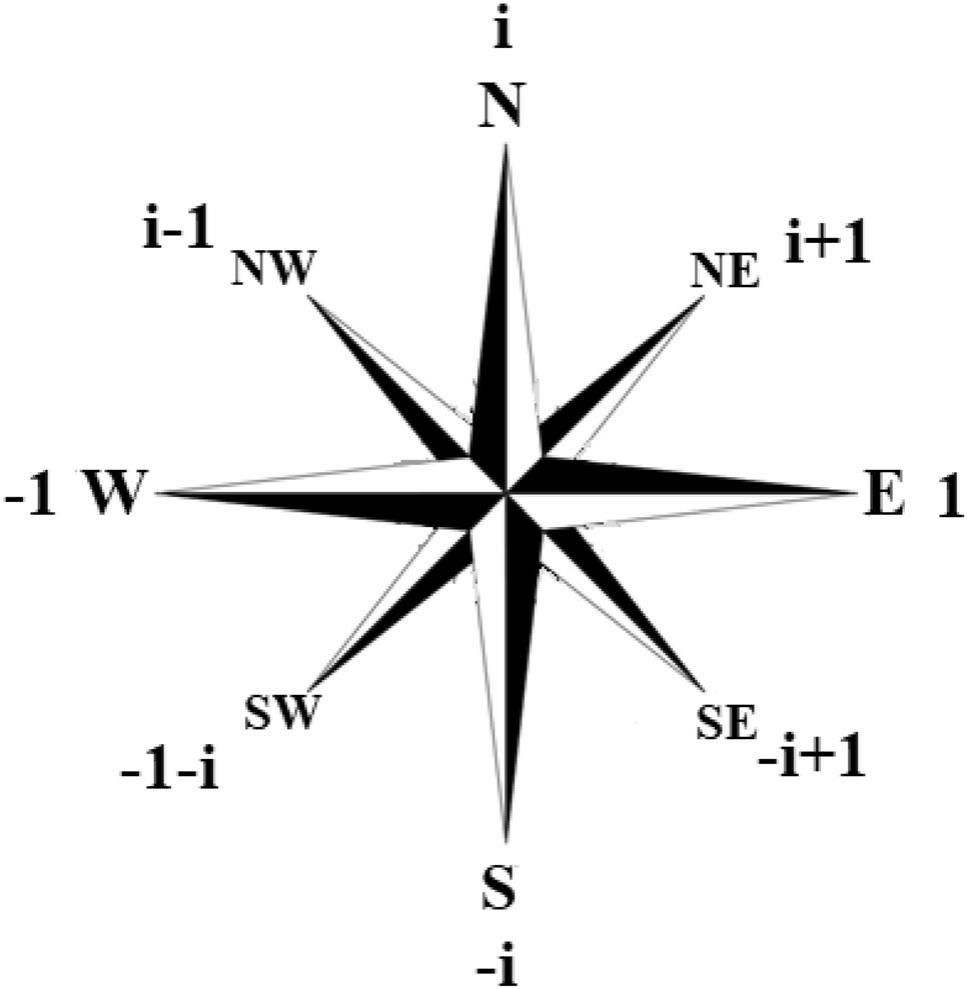
8-wind compass rose (Image was generated in Paint 22H2 https://apps.microsoft.com/store/detail/paint/9PCFS5B6T72H?hl=ru-ru&gl=ru).

It is interesting to note that linguistic variables were introduced in practice long before fuzzy logic was created. For example, in maritime, there has traditionally been a set of commands "full astern, … slow astern, …, slow ahead, …, full ahead." A similar conclusion is also valid in relation to the compass rose (Fig. 1), which is also traditionally used in maritime affairs.

Figure 1 emphasizes that the 8-element compass rose can be used to visually interpret the variables of 9-digit logic.

The variables of such a logic can be put in correspondence with elements of the Galois field $GF({3}^{2})$, which, in turn, can be constructed as an algebraic extension of the field $GF(3)$.

Recall that the method of algebraic extensions can be viewed as a generalization of the method by which complex numbers are constructed. Let us demonstrate the fact on a simple example of the construction of the field $GF({3}^{2})$.

The field $GF(3)$ contains three elements. They can be chosen as $(-\mathrm{1,0},1)$ by setting the following addition rules.

1 $$1+1=-1; -1-1=1$$

According to the method of algebraic extensions, an additional element $\theta$, which is the root of an equation irreducible (having no solutions) in this field, is attached to this (or any other) field.

2 $$f\left(x\right)=0$$

where $f\left(x\right)$ is a polynomial of degree n, $x$ is a variable that takes values in $GF(3)$.

In the special case where $n=2$ such irreducible equation is the equation that allows one to construct complex numbers

3 $${x}^{2}+1=0$$

Then the element $\theta$ can be treated as a logical imaginary unit, and the elements of the field $GF({3}^{2})$ can be represented as

4 $$A={a}_{0}+i{a}_{1},$$

where variables ${a}_{0},{a}_{1}$ belong to the main field.

In this case, we can perform algebraic operations with elements of the form (4) according to formulas (1) and (3). For example,

5 $$i+i=-i; -i-i=i; -i+i=i-i=0,$$

The rules of multiplication remain the same as in the classical use of complex numbers, in particular,

6 $${i}^{2}=-1; i\cdot \left({a}_{1}+i{a}_{2}\right)=i{a}_{1}-{a}_{2},$$

The elements of this field are listed in Table 1.

**Table 1:** Elements of the Galois field $GF({3}^{2})$ in the used representation.

*a*	${a}_{2}=-1$	${a}_{2}=0$	${a}_{2}=1$
${a}_{1}=-1$	$-1-i$	$-1$	$-1+i$
${a}_{1}=0$	$-i$	0	*i*
${a}_{1}=1$	$1-i$	1	$1+i$

In general, any element of the field $GF({3}^{n})$ can be represented as a linear combination of powers of $\theta$.

7 $$A=\sum_{0}^{n-1}{\theta }^{j}{a}_{j}$$

where $\theta$ is a primitive element, ${a}_{j}$ are coefficients from the main field of $GF\left(3\right)$, and $n$ is the degree of the polynomial $f\left(x\right)$ generating the element $\theta$.

The field $GF({3}^{2})$ contains eight non-zero elements (Table 1). Using the notation (4) as a logical coordinate representation, these eight elements can be assigned to the directions of compass roses, which is shown in Fig. 1.

In this example there is a one-to-one correspondence between the elements of multivalued logic, linguistic variables, and elements of the Galois field. More precisely, the elements of the compass rose allow all the above interpretations, which are in a mutually unambiguous correspondence.

Thus, the problem of interpreting multivalued logic variables can be removed if these variables are interpreted through correspondence to fuzzy logic variables. Such an approach, as shown in Section "Reduction of multivalued logic operations to algebraic ones", is generalizable. Namely, in this interpretation, rather a wide range of different terms (including philosophical categories) can be used instead of fuzzy logic variables. Obviously, it is not the specific set of sounds or symbols that represent them that gives meaning to natural language words, but the fact that each of these words is built into the overall structure of the language. Therefore, the meaning of terms is actually determined by the connections between them. The "True—False" opposition, which forms the methodological basis of binary logic, is only the simplest form of such a connection.

Let us show that for the case when the number of variables of multivalued logic is equal to a prime number, any operations in such logic can be reduced to operations of addition and multiplication in the Galois field.

## Reduction of multivalued logic operations to algebraic ones

The operations of multivalued logic are usually displayed in the form of truth tables. So, the following Table 2 are reflecting the operations of the logic of paradoxes by G. Priest^21^.

**Table 2:** Values of the logical function corresponding to the operations of disjunction and conjunction in the logic of paradoxes by G. Priest.

$F\left(x,y\right)= \vee$	*y* = 0	*y* = 1	*y* = 2
$x=0$	0	1	2
$x=1$	1	1	2
$x=2$	2	2	2

$F\left(x,y\right)= \wedge$	*y* = 0	*y* = 1	*y* = 2
$x=0$	*0*	*0*	*0*
$x=1$	*0*	*1*	*1*
$x=2$	*0*	*1*	*2*

In these tables, symbols "0", "1" and "2" are denoting logical variables. The interpretation of the variables of ternary logic as "Truth", "False", "Uncertainly" dates back to the works of Lukasiewicz. The interpretation of such operations (disjunction, conjunction, negation, etc.) as applied to ternary logic can be different, likewise, the use of specific symbols in such tables is nothing more than a matter of agreement.

Such a tabular representation is not always convenient. Operations on logical variables, to which elements of the Galois field are assigned, can be reduced to algebraic ones. For clarity, this can be done, for example, as follows.

To avoid cluttering the notes, we will consider the case of an arbitrary function $f(x,y)$, taking values in the field $GF(p)$, where $x,y$ are elements of the same Galois field. This function corresponds to a truth table given by an ordered enumeration of elements $f({x}_{i},{y}_{j})$, $i,j=\mathrm{0,2}\dots p-1$.

Consider the following expression

8 $${g}_{i}\left(x\right)=1-{\left(x-{x}_{i}\right)}^{p-1}$$

where ${x}_{i}$ is a fixed element of the field $GF(p)$.

It is known from Galois field theory that all nonzero elements of the field $GF(p)$ are roots of the equation

9 $${\theta }^{p-1}-1=0$$

That is, any nonzero element of the field $GF(p)$, if raised to the $p-1$ st power, gives one.

Consequently, the functions ${g}_{i}\left(x\right)$ have the following property

10 $${g}_{i}\left(x\right)=\left\{\begin{array}{c}1, x={x}_{i}\\ 0, x\ne {x}_{i}\end{array}\right.$$

This allows us to treat them as a logical analogue of the δ-function.

Let us form the following polynomial

11 $$F\left(x,y\right)=\sum_{i,j=0}^{i,j=p-1}f\left({x}_{i},{y}_{j}\right){g}_{i}\left(x\right){g}_{j}\left(y\right)$$

where the values $f({x}_{i},{y}_{j})$ form a truth table like the Table 2.

When a particular pair of ${x}_{{i}_{0}},{y}_{{j}_{0}}$ values of logical variables (or more exactly, their corresponding Galois field elements) is substituted into expression (11), all summands appearing in the sum in the right part of formula (11) turn to zero because of relation (8) except the summand for which $i={i}_{0},j={j}_{0}$ is satisfied. Hence, it follows that

12 $$F\left({x}_{{i}_{0}},{y}_{{j}_{0}}\right)=f\left({x}_{{i}_{0}},{y}_{{j}_{0}}\right)$$

We see that the polynomial (11) performs the same functions for multivalued logic as the Zhegalkin polynomial for binary logic, i.e., relation (11) indicates a specific algebraic function which realizes a given truth table. It is also seen that relation (11) admits a generalization to the case of an arbitrary number of logical variables.

Note that control methods based on fuzzy logic are currently being actively developed^22,23^. There are known works, in which such methods are proposed to be used for correcting the course of ships^24^.

Obviously, if a one-to-one correspondence is established between linguistic variables and Galois field elements, then all "commands" and "data" transformed to such variables can be further processed using algebraic functions, which can be constructed knowingly by the method described above.

Of course, for real problems, the number of variables corresponding to an 8-element compass rose is insufficient, but this is not an obstacle.

For example, starting from the field *GF*(7), the elements of which can be chosen as $(-3,-2,-\mathrm{1,0},\mathrm{1,2},3)$, we can construct the field $GF({7}^{2})$.

The elements of this field are also representable in the "two-coordinate" form (4), where the coefficients ${a}_{0}, {a}_{1}$ belong to the field *GF*(7).

The entry (4) in this case, for clarity, can be interpreted, for example, as a discrete representation of the velocity vector (in the plane), which fully corresponds to the traditional complex representation of vectors. The difference is that using the field $GF({7}^{2})$, the velocity components are discrete, and they can be assigned to seven linguistic variables "full astern, half astern, small astern, stop engine, small ahead, half ahead, full ahead".

**Figure 2:**
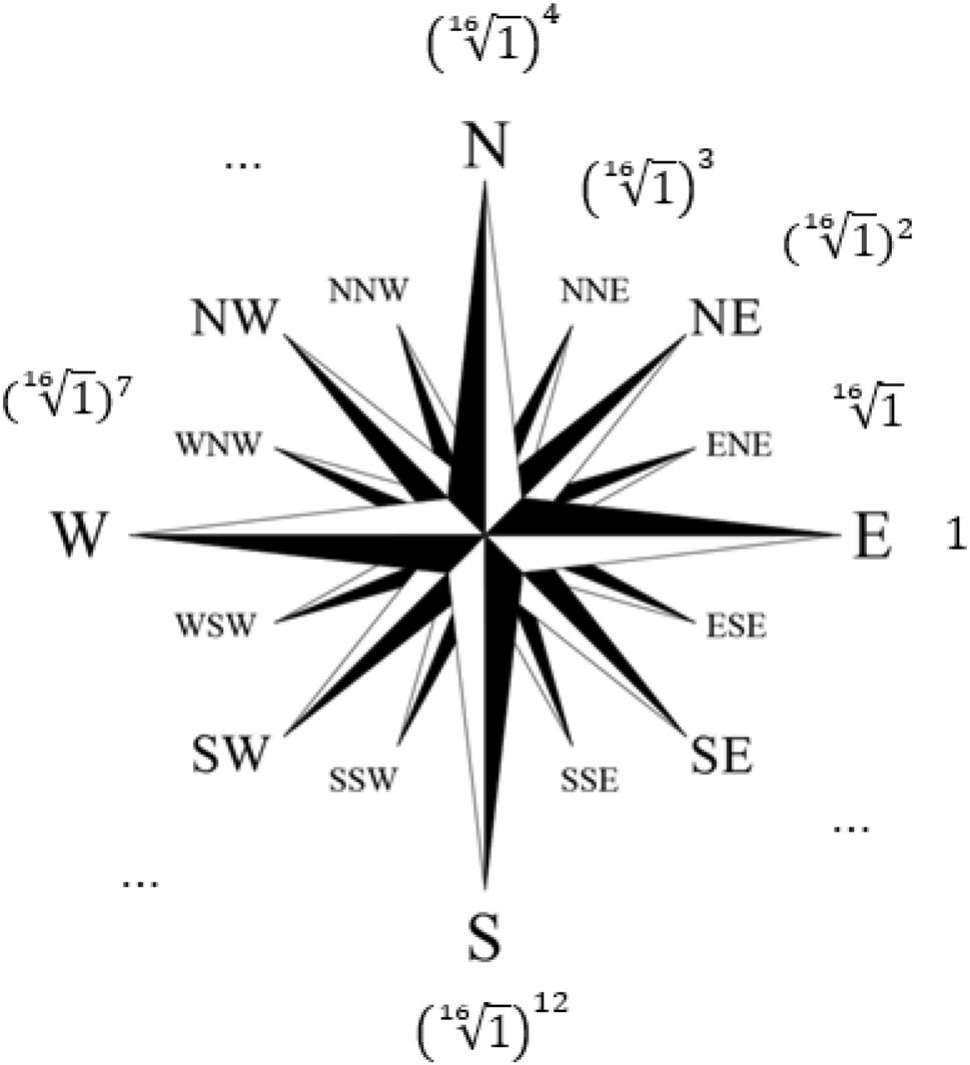
16-element compass rose (Image was generated in Paint 22H2 https://apps.microsoft.com/store/detail/paint/9PCFS5B6T72H?hl=ru-ru&gl=ru).

The use of such a field also allows us to map the linguistic variables corresponding to the 16-item compass rose, Fig. 2.

Namely, the number of non-zero elements of the field $GF({7}^{2})$ is 48. Consequently, they are all roots of the equation

13 $${x}^{48}-1={\left({x}^{16}\right)}^{3}-1=\left({x}^{16}-1\right)\left({x}^{32}+{x}^{16}+1\right)=0$$

Formula (13), among other things, shows that among the elements of the field $GF({7}^{2})$ there are 16 elements that satisfy the equation

14 $${x}^{16}-1=0$$

These 16 elements can be viewed as roots of the 16th degree from one, and they form a group by multiplication. Consequently, they can be assigned linguistic variables corresponding to the 16-element compass rose.

Thus, the mutually unique correspondence between multivalued ${p}^{n}$-logics, where $p$ is prime number, $n$ is integer and Galois fields $GF({p}^{n})$ creates all preconditions for making operations on variables of multivalued logic as clear as possible.

It can be argued that visualization in this respect is provided not so much for variables of multivalued logic as for elements of Galois fields. However, the visual representation of operations on the variables of multivalued logic mapped through Galois fields has also a philosophical aspect directly related to the problem of interpretation of the values of the mentioned variables and to the problem of correlation of laws of thinking and multivalued logics touched upon in^8^.

Namely, the meaning of the variables of binary logic relates to the philosophical category of truth. This category belongs to the number of basic concepts, the question about the nature of which is closely related to the problem of the existence of undefined concepts. Indeed, to "define" means to reveal the meaning of one term through others. Trying to reveal all the terms available in a language in this way leads knowingly to a vicious circle.

Objective dialectics finds a way out by defining the basic categories through the oppositions "quantity–quality," "content–form," etc. Such an approach, in particular, was used^13,25^ in order to reveal as correctly as possible, the meaning of the category "information", which it was suggested to consider as a philosophical category paired with the category of matter.

The problem of adequate interpretation of the concept "information" as emphasized in^26,27^ becomes more and more relevant in connection with the research in the field of artificial intelligence, but for the purposes of our article the approach of "definition through contraposition" itself is more important.

Namely, it shows that for the definition of basic notions the most important is the structure of relations between them, and contraposition is only one of the forms of such relations, and the one that knowingly corresponds to binary logic and Galois binary fields. Obviously, other forms of connections between basic concepts cannot be reduced to a simple contraposition.

This indicates for example the existence of a pronounced methodological (philosophical) aspect of the development of command languages (even at the level of specific technical systems), which constitute a closed whole at the expense of relations written in algebraic form. Moreover, it is extremely difficult to develop closed "language" systems at the level of abstraction. It is much more convenient (and illustrative) to do this by solving specific problems, for example, those related to control of moving vehicles, in terms of fuzzy logic converted into algebraic form.

This formulation of the question makes it even more urgent to ensure the visibility and usability of multivalued logics. The following section deals with specific computational tools oriented to the use of logics corresponding to the fields $GF({7}^{n})$.

This example allows you to clearly demonstrate that it is possible to implement various kinds of devices that perform calculations in terms of multivalued logic, but at the same time built on the basis of typical electronic components using binary logic.

## Computational implementation of seven-digit logic operations

Currently, algorithms and schemes of radioelectronic devices that perform calculations modulo are widely represented in the literature. Thus, such algorithms are used in encryption, coding devices, in compression and transmission of information, in automation devices^28–30^.

As shown above, any functions whose arguments are variables taking values in the Galois field can be explicitly reduced to algebraic expressions which involve only multiplication and addition operations modulo $p$.

Consequently, multipliers and adders modulo $p$ are the basis for automating any operations on logical (linguistic) variables. Devices of this type can be implemented by rather simple means, as it is proved below.

**Figure 3:**
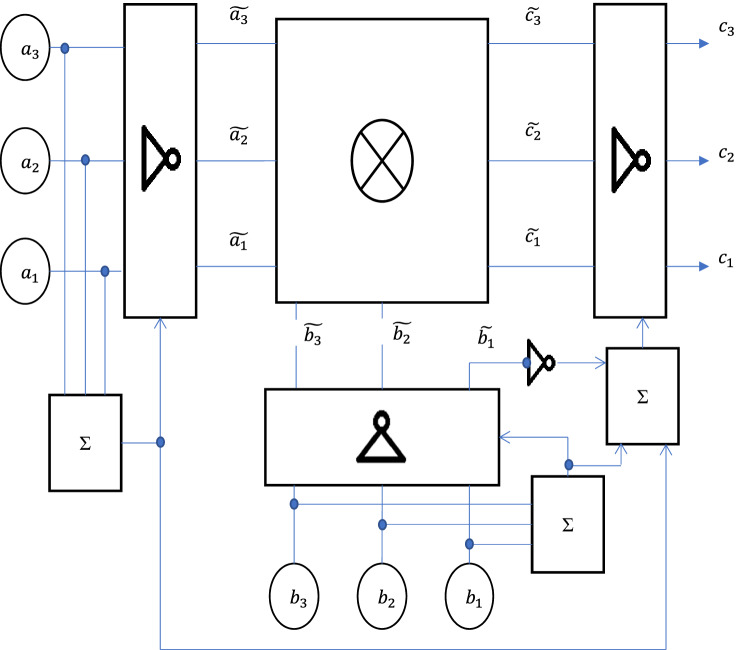
Block diagram of the modulo multiplier by seven (Image was generated in PowerPoint Microsoft 365 https://www.microsoft.com/en-ww/microsoft-365/powerpoint).

The block diagram of the multiplier of the considered type is presented in Fig. 3. The scheme includes adders (marking on the scheme is $\Sigma$), which count the number of units on the inputs ${\mathrm{a}}_{i}$ corresponding to the number representation in binary form. It is supposed, that on the input of the system no signals corresponding to number 7 or number 0 are input. This is acceptable, since when calculating modulo 7, $7\equiv 0(7)$ takes place, therefore, in this case, the calculated product is equal to zero. In this case $\Sigma {\mathrm{a}}_{i}$ can take values 1 or 2, as in the binary notation of numbers that vary from 1 to 6, there are at least one and at most two units.

Then

15 $$6\cdot {a}_{3}{a}_{2}{a}_{1}{=}_{(7)}{\overline{a} }_{3}{\overline{a} }_{2}{\overline{a} }_{1}$$

where *a*_*i*_ are characters in the binary notation of the number, a bar over the character means the inversion operation, i.e., 0 changes to 1 and vice versa.

Due to the associativity of multiplication modulo, the product of any two non-zero elements of the field *GF*(7) can be reduced to the multiplication of two numbers in binary representation, and in both of these numbers only one of the symbols *a*_*i*_ will be non-zero.

Correspondent operation is realized by the inverter block (the standard inverter designation is used in the scheme) controlled by the signal taken from $\Sigma$ elements. If logical zero is formed on the output of these elements, signals ${\mathrm{a}}_{i}$ and ${\mathrm{b}}_{i}$ remain unchanged, if logical one, they take inverse values.

The signal sets ${\widetilde{\mathrm{a}}}_{i}$ and ${\widetilde{\mathrm{b}}}_{i}$, reduced to a format in which only one of the variables of these sets is non-zero, are fed to the direct multiplier block (schematic designation—⊗).

The signal set ${\widetilde{c}}_{i}$ from the output of the direct multiplier is fed to the output inverter block, which operates in the same way as the input inverter block.

**Figure 4:**
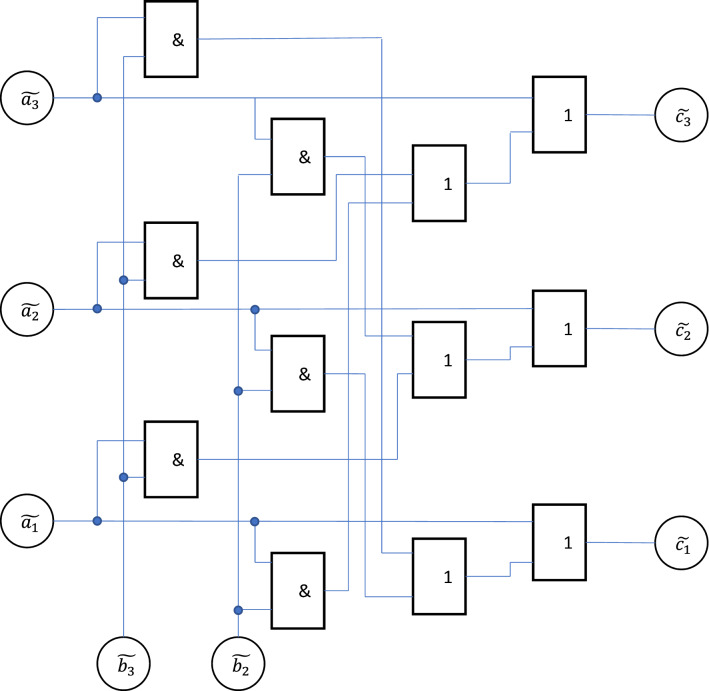
Schematic diagram of a direct multiplier (Image was generated in PowerPoint Microsoft 365 https://www.microsoft.com/en-ww/microsoft-365/powerpoint).

The schematic diagram of the direct multiplication block is shown in Fig. 4.

This block works as follows.

The prime number 7 is a special case of prime Mersenne numbers, represented in the form ${p}_{m}={2}^{n}-1$. Such numbers have the following property. Multiplication of any number by 2 modulo ${p}_{m}$ results is a cyclic permutation of symbols. For example,

16 $$2\cdot {a}_{2}{a}_{1}{a}_{0}{=}_{(7)}{a}_{1}{a}_{0}{a}_{2}$$

where ${a}_{i}$ are binary characters.

Let us consider the product of two numbers $B\cdot A$ written in binary notation. We have

17 $$B\cdot A{=}_{\left(2\right)}{b}_{3}\cdot {2}^{2}\cdot A+{b}_{2}\cdot {2}^{1}\cdot A\cdot +{b}_{1}\cdot {2}^{0}\cdot A$$

According to formula (16), products ${2}^{m}\cdot A$ may be written through cyclic permutations, i.e. the product calculated modulo 7, is the sum of the following three numbers written in binary form as

18 $${b}_{1}\cdot {a}_{3}{a}_{2}{a}_{1}$$

19 $${b}_{2}\cdot {a}_{2}{a}_{1}{a}_{3}$$

20 $${b}_{3}\cdot {a}_{1}{a}_{3}{a}_{2}$$

where only one of the ${b}_{i}$ values is 1, and the rest are 0.

Each of the binary three-digit numbers appearing in formulas (18)–(20) can also be written in powers of two.

Consequently, the result of multiplication in calculations modulo 7 can be written as

21 $$B\cdot A{=}_{\left(2\right)}{c}_{3}\cdot {2}^{2}+{c}_{2}\cdot {2}^{1}\cdot +{c}_{1}\cdot {2}^{0},$$

where

22 $${c}_{3}={b}_{1}{a}_{3}+{b}_{2}{a}_{2}+{b}_{3}{a}_{1}$$

23 $${c}_{2}={b}_{1}{a}_{2}+{b}_{2}{a}_{1}+{b}_{3}{a}_{3}$$

24 $${c}_{1}={b}_{1}{a}_{1}+{b}_{2}{a}_{3}+{b}_{3}{a}_{2}$$

Since inverters are used in the circuit under consideration, in formula (22)–(24) only one of the values ${a}_{i}$ and only one of the values ${b}_{i}$ is equal to 1, the rest are equal to 0. Consequently, among all the values ${c}_{i}$ only one is also equal to 1, and the rest are 0.

Therefore, the result of the product corresponds to the three outputs of the circuit, on which the logical variables ${c}_{i}$ are formed.

Since of all the values ${b}_{i}$ only one is equal to 1, then three options are possible.

If $${b}_{1}=1$$, then

25 $$\left({c}_{3},{c}_{2},{c}_{1}\right)=\left({a}_{3},{a}_{2},{a}_{1}\right)$$

If $${b}_{2}=1$$, then

26 $$\left({c}_{3},{c}_{2},{c}_{1}\right)=\left({a}_{2},{a}_{1},{a}_{3}\right)$$

If $${b}_{3}=1$$, then

27 $$\left({c}_{3},{c}_{2},{c}_{1}\right)=\left({a}_{1},{a}_{3},{a}_{2}\right)$$

If ${b}_{1}=1$, then the state of outputs c_i repeats the state of inputs ${a}_{i}$, if ${b}_{2}=1$, then there is a cyclic permutation one position to the right, and if ${b}_{3}=1$, then one position to the left.

A scheme that provides such a permutation can be implemented in various ways. One of them is based on a set of operations, which can be represented schematically as follows.

28 $$NO \left[\left(\mathrm{0,1},0\right) OR \left(\mathrm{0,0},1\right)\right]\to NO \left(\mathrm{0,1},1\right)\to \left(\mathrm{1,0},0\right)$$

29 $$\left(\mathrm{0,1},0\right) OR \left(\mathrm{0,0},0\right)\to \left(\mathrm{0,1},0\right)$$

These notations imply that the $NO$ and $OR$ operations are applied to each of the boolean variables appearing in the sequences. Formulas (28) and (29) show only a particular case; they obviously remain valid for cyclic permutations as well.

From these formulas it follows that the permutation corresponding to formulas (25)–(27) can also be implemented in the way that is implemented by the scheme of Fig. 4.

In this case, if ${b}_{1}=0$, an additional inversion of the signal is performed, which corresponds to the execution of operation (28). According to the diagram in Fig. 4 this operation is performed by the adder, the output of which is connected to the output inverter.

**Figure 5:**
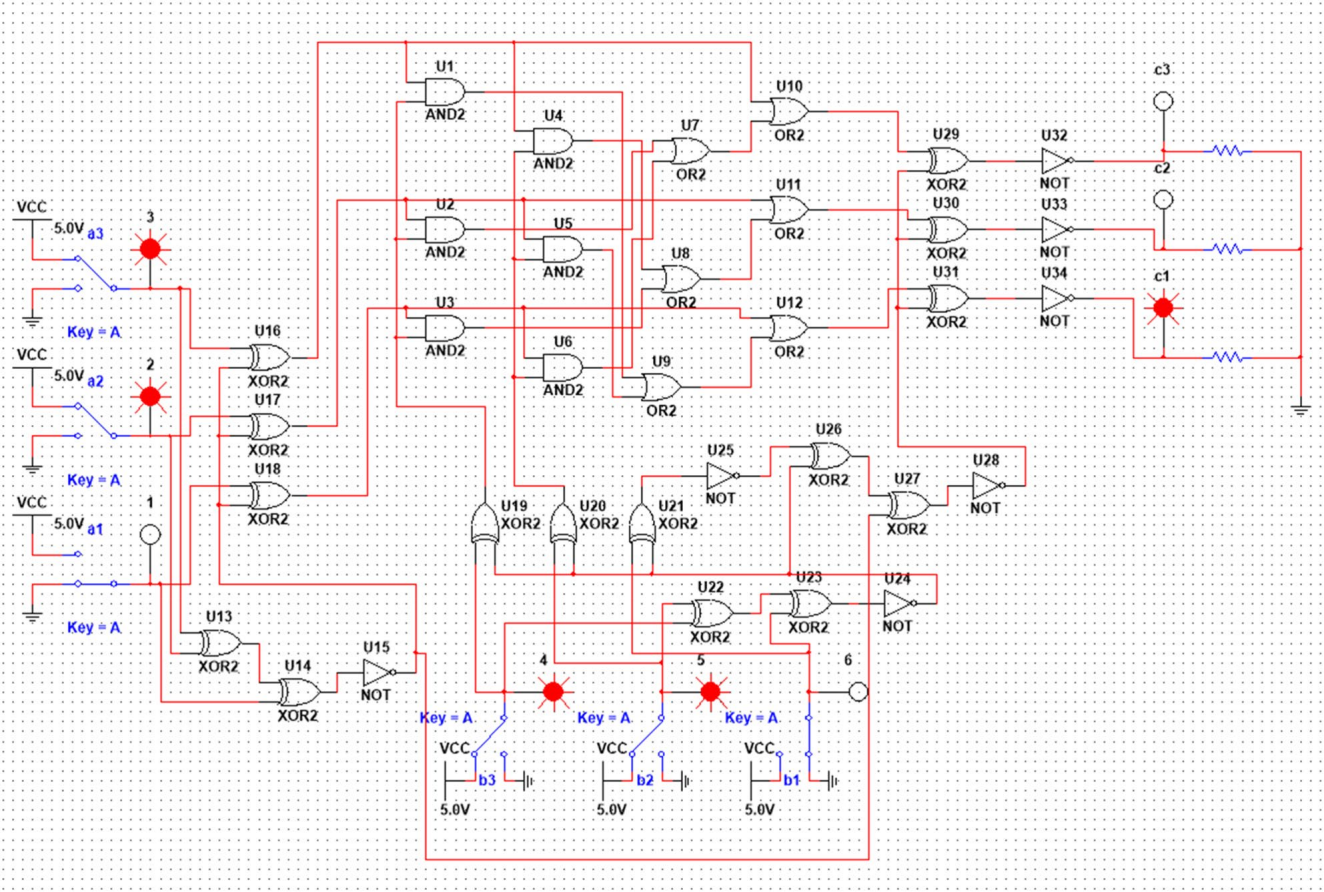
Multiplier circuit modulo seven, assembled in the NI Multisim program. (Image was generated in NI Multisim 14.1 https://www.ni.com/ru-ru/support/downloads/software-products/download.multisim.html#306441).

The complete scheme, made in the NI Multisim application^31^, is shown in Fig. 5.

As follows from the above description of the multiplier, its scheme takes into account the most important specific features of computational systems carrying out operations in Galois fields, which are directly connected with operations of multivalued logic.

## Conclusion

This paper shows that the problem of interpreting the variables of multivalued logic does not necessarily have to be solved through the involvement of the philosophical category of truth. A possible option is to use a close connection between the variables of multivalued logics, whose number of elements is equal to the degree of a prime number, with Galois fields. In this case it is possible, among other things, to establish a connection between the variables of multivalued logic and the linguistic variables used in fuzzy logic. In addition, this relationship allows to reduce any operations on variables of multivalued logic to the calculation of algebraic functions whose arguments take a value in the Galois field. Otherwise, any operations of multivalued logics of the specified type can be reduced to the operations of addition and multiplication modulo the degree of a prime number.

Such operations, in their turn, can be realized by means of radio electronic circuits assembled on typical elements, performing operations of binary logic. At the same time, as shown in the example of implementation of such circuits, they can be quite simple.

## Data Availability

All data generated or analysed during this study are included in this published article.
